# 3D printing of multicolor luminescent glass†

**DOI:** 10.1039/c8ra06706f

**Published:** 2018-09-10

**Authors:** Chang Liu, Bin Qian, Rongping Ni, Xiaofeng Liu, Jianrong Qiu

**Affiliations:** School of Materials Science and Engineering, Zhejiang University Hangzhou Zhejiang China; State Key Laboratory of Modern Optical Instrumentation, School of Optical Science and Engineering, Zhejiang University Hangzhou Zhejiang China qjr@zju.edu.cn

## Abstract

The development of the stereolithography technique for the additive manufacturing of silica glass has brought revolutionary change in glass manufacturing. Here, we demonstrate the fabrication of 3D luminescent transparent glass parts manufactured by the stereolithographic technique together with solution impregnation and high temperature sintering. Prefabricated glass parts with nanopores were prepared by the stereolithography technique and debinded and pre-sintered at first. To functionalize the additive manufactured glass with photoluminescence, Eu^3+^, Tb^3+^ and Ce^3+^ ions were doped with a solution impregnation method and further sintered at high temperature. The photoluminescence from these rare earth ions in the blue, cyan and red spectral region can be facilely generated by illumination with a 254 nm UV lamp. Furthermore, we developed a space-selective doping method that enables the doping of different ions in different parts of a silica glass in a space-selective fashion, resulting in a multicolor luminescent glass object giving distinguishable luminescence from each part.

## Abstract 2

A novel technique enables the design of both the shape and the function of single glass devices.

## Graphical abstract

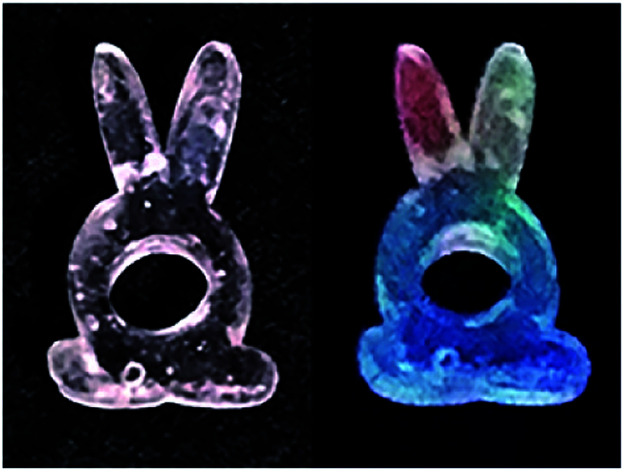

## Introduction

Glass is an important material used in laboratory, industry and our daily life for its transparency and superior chemical and thermal resistance, which make glass irreplaceable by other materials. However, glass is hard and brittle which makes it extremely difficult for shaping using traditional processing technologies, and this limits the applications of this material.^1–4^ Until now, a number of additive manufacturing (which is also designated as 3D printing) technologies for glass have been developed which brought more flexibility in shaping this material.^5–11^ Among these techniques, stereolithography is the most reliable one due to its higher resolution and lower risk of damage,^6,11^ and this has potential applications for microfluidics, optical lenses and delicate artifacts.^12,13^

The shape is not the only requirement for glass devices; sometimes, optical properties, mechanical properties and bioactivities are required for particular applications. Photoluminescence (PL) is one of the most important properties of glass which is normally introduced through the doping of luminescent ions. Photoluminescent glasses have important applications in white light emission, laser generation and optical temperature sensing.^14–16^ To dope ions in the process of glass additive manufacturing with stereolithography, the method by impregnating the porous glass with solutions containing the target ions followed by sintering is quite suitable as porous glass is the intermediate product in glass additive manufacturing with stereolithography.^17–20^

In this work, Eu^3+^, Ce^3+^ and Tb^3+^ doped silica glass objects were manufactured with a top-down stereolithographic technique combined with a doping process by solution impregnation. The formation of the dense silica glass was confirmed by scanning electron microscopic (SEM) images, X-ray diffraction (XRD) and Raman spectra. The doped glass showed the characteristic emission of Eu^3+^, Ce^3+^ and Tb^3+^ ions under the excitation at 254 nm which was confirmed by the PL spectra. Furthermore, we developed a space-selective doping method which dopes different ions in different parts of a glass device. This technique will have significant implications for glass manufacturing that not only the shape but also the function of a single glass device can be designed, which has great potential for applications in integrated optics.

## Experimental

2-Hydroxyethyl methacrylate (HEMA, 99%), poly(ethylene glycol) diacrylate 200 (PEGDA 200), diethyl phthalate (99.5%), 4-methoxyphenol (MEHQ, 99%), 2,2-dimethoxy-2-phenylacetophenone (DMPA, 99%), Eu(NO_3_)_3_·6H_2_O (99.9%), Ce(NO_3_)_3_·6H_2_O (99.9%) and Tb(NO_3_)_3_·5H_2_O (99.9%) were provided by Aladdin, China. Amorphous silica nanoparticles (Aerosil OX50) were provided by Evonik, Germany. Tinuvin 1130 was provided by Basf, Germany. Ethanol (99.7%) was provided by Sinoreagent, China.

28.1 wt% HEMA (the reactive monomer), 14.5 wt% diethyl phthalate (the plasticizer) and 3.7 wt% PEGDA 200 (the crosslink agent) were mixed to form a homogeneous solution. 53.7 wt% amorphous silica nanoparticles were added to the solution in 50 doses, and were dispersed with a dissolver (D500, Dragonlab) after each dosing. Afterwards, 0.4 wt% DMPA (the photo initiator) and 0.2 wt% Tinuvin 1130 (the photo absorber) were dispersed into the mixture.

**Fig. 1:**
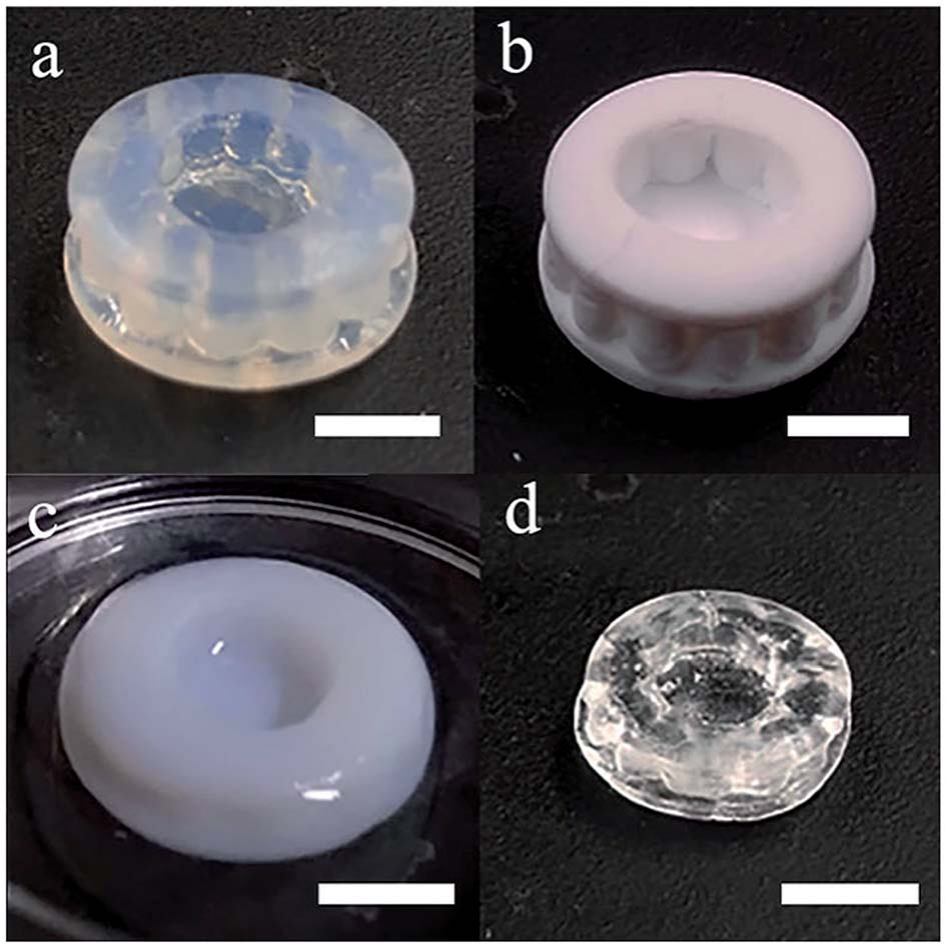
Photos of (a) the photopolymerized silica-polymer composite, (b) the porous silica glass after debinding, (c) the porous silica glass immersed in ethanol solution of RE ions. (d) The dense silica glass with RE ions doping after sintering. The scale bars represent 5 mm.

The additive manufacturing process with a top-down stereolithography system was reported in our previous work.^11^ The hatch distance was set to 70 μm, the laser power was set to 145 mW and the scan speed was set to 60 mm s^−1^. The additive manufactured silica-polymer composites (Fig. 1a) were debinded and pre-sintered at 1000 °C (with the temperature curve shown in Fig. S1a†) in a muffle furnace (KSL-1100X, Kejing Materials Technology, China). The obtained porous glass objects (Fig. 1b) were immersed in 0.005 mol L^−1^ Eu(NO_3_)_3_, Ce(NO_3_)_3_, or Tb(NO_3_)_3_ ethanol solutions (Fig. 1c) and were kept in these solutions for 15 minutes. These objects were dried at 60 °C in an electric oven with constant air flow for 1 hour (BPG-9040A, Yiheng, China), and then they were moved to a tube furnace (GSL-1400X, Kejing Materials Technology, China) with a corundum tube and were sintered at 1250 °C for 3 hours (with the temperature curve shown in Fig. S1b†) in vacuum. Silica glass objects (Fig. 1d) with the same shapes were obtained, and the linear shrinkages were around 27.0%. For space-selective doping, droppers were used to drip different solutions on different parts of a porous glass object. The solutions were driven to the body from the surface by capillary forces. Afterwards, the object was dried and sintered in the same way as the normally doped glass.

The emission spectra of the doped silica glass under the excitation at 254 nm were measured with a fluorescent spectrometer (FLSP920, Edinburgh Instruments, Britain). The photoluminescent photos were shot under the illumination of a 254 nm UV lamp in a dark room with a smart phone, and the exposure time was set to 1/15 s. Pore distribution and porosity of the porous silica glass were measured with a nitrogen adsorption instrument (ASAP 2020 HD88, Micromeritics, US). X-ray diffraction patterns of the powdered doped silica glass samples were measured with an X-ray diffraction spectrometer (D/MAX 2550/PC, Rigaku). Raman spectra of the doped silica glass were measured with a Raman spectrometer (LabRam HR UV, Jobin-yvon, France) with a 514 nm laser. The SEM images were taken with a scan electron microscope (Ultra 55, Carl Zeiss Jena, Germany), and a layer of gold was sputtered to the surface of the sample to enhance the conductivity.

## Results and discussions

**Fig. 2:**
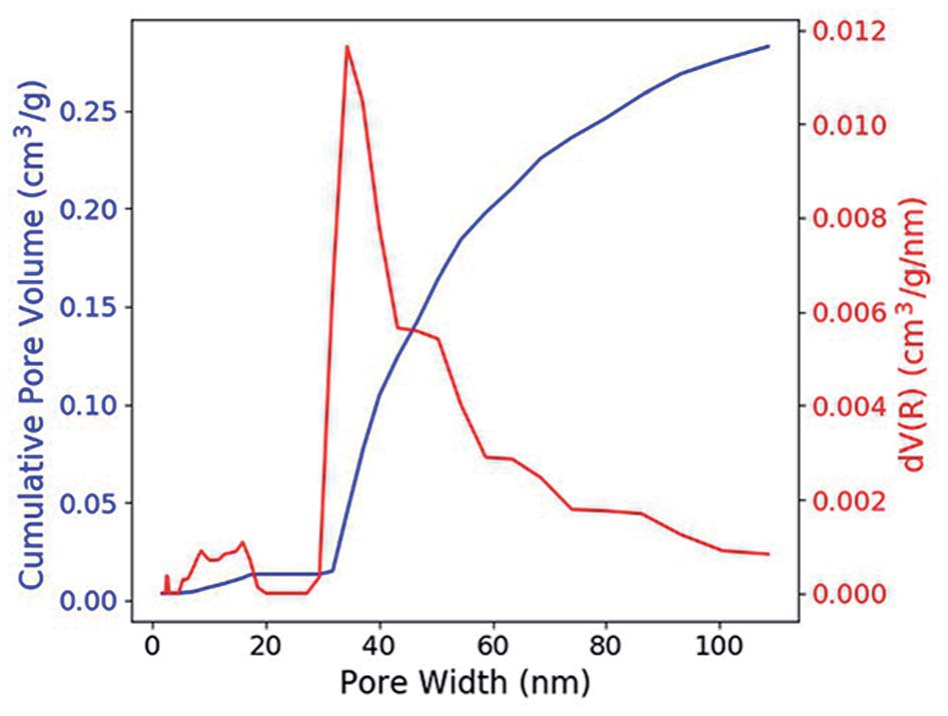
Cumulative pore volume and the pore width distribution of the porous silica glass.

According to the nitrogen adsorption and desorption experiment (Fig. 2), most of the pores have the width between 30 nm and 50 nm and the average value is 31.5 nm. The pores provide enough capillary force that drive the solution to fill these pores. With the porosity, which is 0.316 cm^3^ g^−1^ (41.0 vol%), the concentration of the RE dopant can be estimated with the formula,

1 *c*_g_ = *c*_s_*Pρ*_g_

where *c*_s_ is the RE concentration of the solution, *P* is the porosity and *ρ*_g_ is the density of the silica glass which is 2.20 g cm^−3^. The concentration of RE ions in the doped glass is found to be 3.48 × 10^−3^ mmol cm^−3^.

**Fig. 3:**
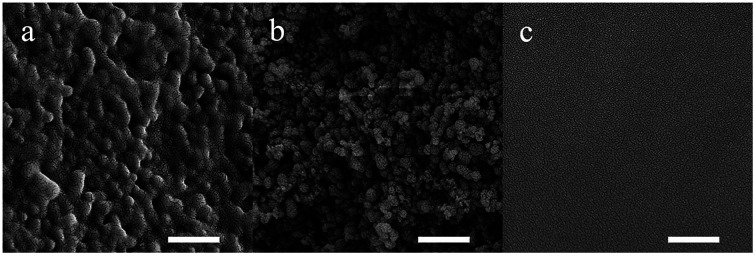
SEM images of the (a) silica-polymer composite, (b) porous silica glass and (c) sintered silica glass. The scale bars represent 500 nm.

The SEM images show the transformation in the microstructure from the silica-polymer composite to the sintered silica glass (Fig. 3). Polymers were totally removed during debinding and the porous glass sample looks like an accumulation of silica powders which contains high volume fraction of pores (Fig. 3b). After sintering, the pores disappear and a smooth surface of the sintered glass is observed (Fig. 3c). The disappearance of the pores during sintering results in the formation of dense, transparent silica glass.

**Fig. 4:**
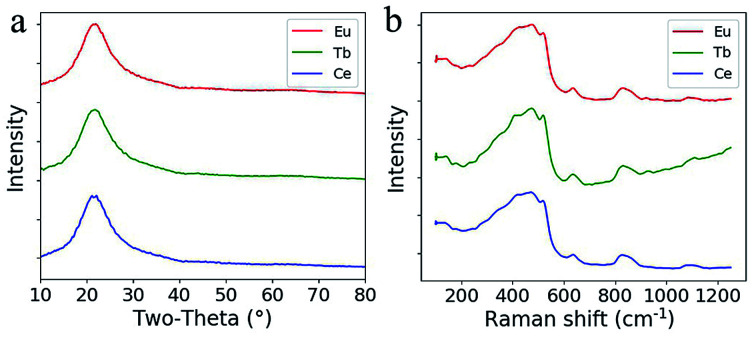
(a) XRD patterns of the doped silica glass. (b) Raman spectra of the doped silica glass.

From the XRD pattern shown in Fig. 4a, the broadened hump centered at around 23° is typical for silica glass and no quartz crystals are formed during sintering after doping with RE ions. The Raman spectra of the doped silica glass are similar to that of the fused silica glass (Fig. 4b). The peaks for the breathing mode from 4 and 3 membered ring structures in the silica network around 490 cm^−1^ and 605 cm^−1^ are observed, which confirm the formation of silica glass after sintering.^21–23^ The rising Raman scattering intensity of the Tb^3+^ doped glass from 700 cm^−1^ to 1200 cm^−1^ comes from the photoluminescence background from Tb^3+^ ions excited by the 514 nm laser.

**Fig. 5:**
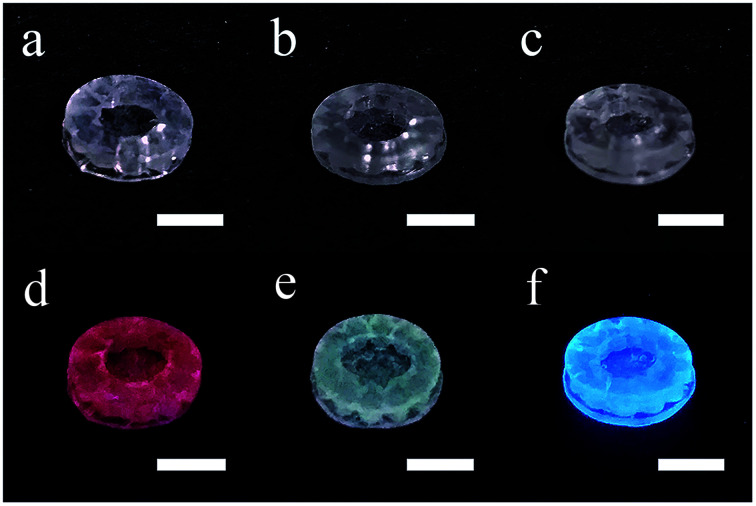
Photos of the additive manufactured silica glass doped with (a) Eu^3+^, (b) Tb^3+^ and (c) Ce^3+^ ions and (d–f) their luminescent performance under a 254 nm UV lamp. The scale bars represent 5 mm.

**Fig. 6:**
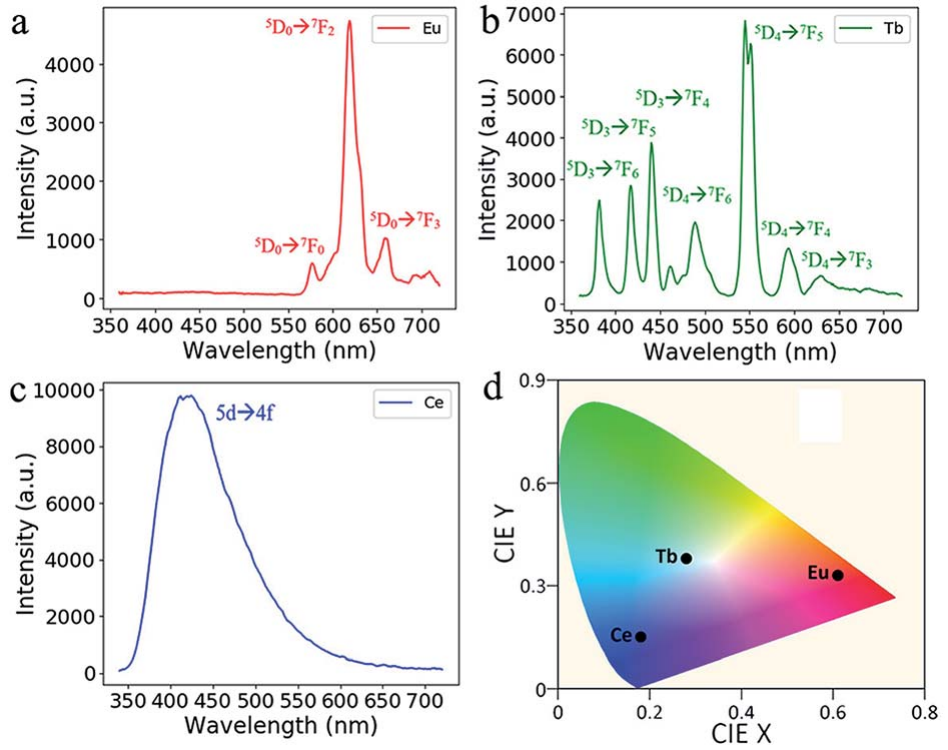
Emission spectra of the additive manufactured glass doped with (a) Eu^3+^, (b) Tb^3+^ and (c) Ce^3+^ ions under the excitation at 254 nm. (d) The CIE coordinate of the RE-doped glasses.

In Fig. 5, the RE doped glass objects look the same under natural light, because Eu^3+^, Tb^3+^ and Ce^3+^ ions don't have intense absorption in the visible range due to their low concentration. However, emissions of the Eu^3+^, Tb^3+^ and Ce^3+^ ions doped glass can be clearly observed under the excitation of a 254 nm lamp. According to the photoluminescence spectra (Fig. 6), the emission peak from the ^5^D_0_ → ^7^F_2_ transition of Eu^3+^ ions at 617 nm dominates the photoluminescence spectra of the Eu^3+^ doped silica glass.^24^ The emission peak of the Ce^3+^ doped silica glass at 350–550 nm is from the 5d → 4f transitions of Ce^3+^ ions.^25^ The emission spectra of Tb^3+^ doped silica glass is much complicated which consists of multiple peaks that distribute from 360 nm to 650 nm. The emission peak at 550 nm from the ^5^D_4_ → ^7^F_5_ transition of Tb^3+^ ions shows the highest intensity among all the peaks.^26^

**Fig. 7:**
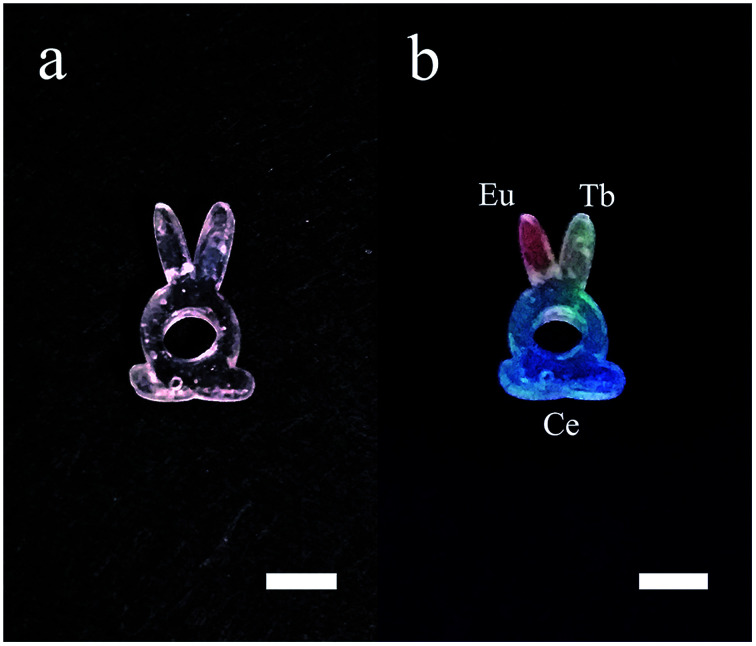
Photos of the additive manufactured glass with each RE ions doped in different parts. (a) Under natural light and (b) under illumination by a 254 nm UV lamp. The scale bars represent 5 mm.

The space-selectively doped glass looks the same as undoped ones under natural light, as the concentrations of the dopants are relatively low. However, under the illumination by a 254 nm UV lamp, the parts that doped with different ions emitted different lights which are distinguishable by eyes (Fig. 7). Furthermore, other rare earth ions can be doped and the spatial resolution for doping can be improved. This space-selective doping technique may have important implications in the manufacturing of photonic glasses in the future.

## Conclusion

Luminescent transparent silica glass doped with RE ions were made by additive manufacturing based on stereolithography and solution impregnation. The presence of RE ions does not impede the formation of silica glass, and all the doped objects could be activated by a 254 nm UV lamp. The Eu^3+^, Tb^3+^ and Ce^3+^ doped glass objects emitted red, cyan, and blue light under the excitation of the 254 nm lamp according to the photoluminescent spectra and the CIE coordinate. Furthermore, we developed a space-selective doping method. Different parts of the space-selectively doped glass emitted different light according to the doping ions. This method may enable the design of both the shape and the function in a single glass device.

## Conflicts of interest

There are no conflicts to declare.

## Supplementary Material

RA-008-C8RA06706F-s001
