# Supplementary material for: 3D printing of multicolor luminescent glass
Source: RSC Adv. 2018 Sep 10;8(55):31564–7. doi: 10.1039/c8ra06706f (PMC9085626; doi:10.1039/c8ra06706f)
Supplement: RA-008-C8RA06706F-s001 [file RA-008-C8RA06706F-s001.pdf]

Electronic Supplementary Material (ESI) for RSC Advances.
This journal is © The Royal Society of Chemistry 2018

Supplementary Information

The temperature curves used in the debinding and sintering processes.

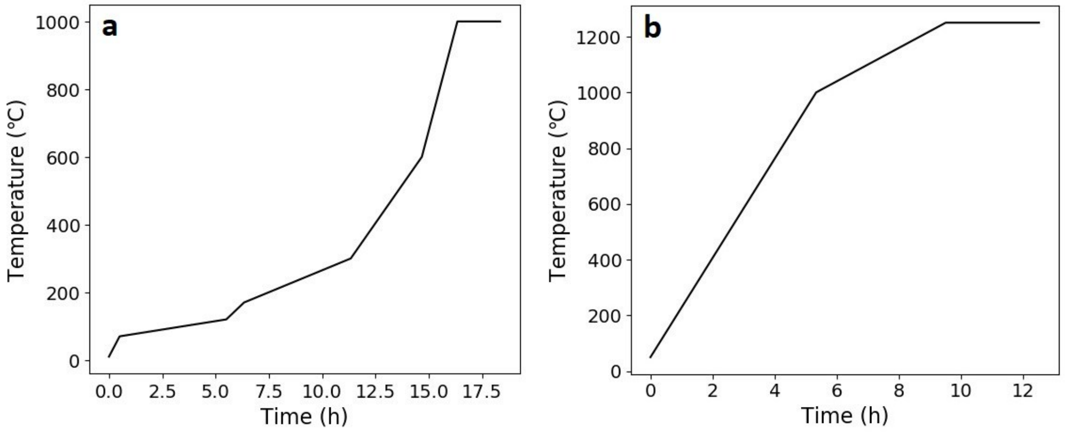

Fig. S1 (a) The temperature curve for debinding to obtain the porous glass (b) The temperature curve for sintering the porous silica glass
